# 1071. Efficacy of Anti-Staphylococcal Lysin, LSVT-1701, in Combination with Daptomycin in Experimental Left-Sided Infective Endocarditis (IE) Due to Methicillin-Resistant *Staphylococcus aureus* (MRSA)

**DOI:** 10.1093/ofid/ofab466.1265

**Published:** 2021-12-04

**Authors:** David Huang, Eric Gaukel, Katyna Borroto-Esoda, Yan Xiong, Wessam Abdelhady, Arnold Bayer

**Affiliations:** 1 Lysovant, Houston, Texas; 2 The Lundquist Institute for Biomedical Innovation at Harbor-UCLA Medical Center, Torrance, California

## Abstract

**Background:**

Anti-staphylococcal phage lysins, such as LSVT-1701, represent important candidate adjunctive agents against invasive MRSA infections because of both their microbicidal and anti-biofilm properties. We, thus, sought to examine the *in vivo* efficacy of LSVT-1701 combination with daptomycin, a standard-of-care anti-MRSA agent with proven efficacy against bacteremia and IE in humans.

**Methods:**

We utilized the rabbit model of aortic valve infective endocarditis (using the prototype MRSA strain, MW2) to examine the combined efficacy of LSVT-1701 plus daptomycin. We examined microbiologic outcomes in distinct target tissues (cardiac vegetations, spleen and kidney) in this model, as well as the pharmacokinetic and pharmacodynamic drivers and target attainment values most predictive of treatment outcomes. LSVT-1701 was given at two dose-regimens (32.5 mg/kg and 50 mg/kg) with different dose-durations (single dose vs daily dose for 2 d vs daily dose for 4 d); daptomycin was administered in combination with daptomycin at a sub-lethal daily dose of 4 mg/kg for 4 d to maximize potential synergistic interaction outcomes.

**Results:**

The Table below shows all LSVT-1701 regimens in combination with daptomycin significantly reduced MRSA burdens in all target tissue as compared to untreated controls. The reduction in MRSA counts was statistically significant in instances of both increasing LSVT-1701 dose level (i.e., single doses of 50 mg/kg vs 32.5 mg/kg iv), as well as increased numbers of lysin doses (i.e., four daily doses vs a single-dose or two daily-doses) in combination with daptomycin. Of note, both the LSVT-1701 50 mg/kg and 32.5 mg/kg daily dose-strategies given for four days in combination with daptomycin sterilized all target tissues (i.e., quantitative cultures ≤ the lower limit of detection of 1 log_10_ CFU/g. tissue).

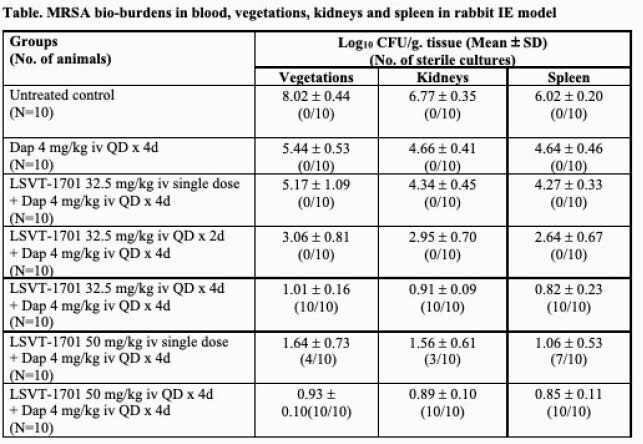

MRSA bio-burdens in blood, vegetations, kidneys and spleen in rabbit IE model

**Conclusion:**

LSVT-1701 administered at 32.5 or 50 mg/kg in a 4 d daily-dose regimen in combination with daptomycin resulted in microbiologic sterilization of all target organs in this MRSA IE model. These data support further clinical development of LSVT-1701 for the treatment of MRSA endovascular infections including IE.

**Disclosures:**

**David Huang, MD, PhD**, **Lysovant** (Consultant) **Eric Gaukel, BS**, **Lysovant** (Employee) **Katyna Borroto-Esoda, PhD**, **Lysovant** (Consultant) **Arnold Bayer, MD, PhD**, **Lysovant** (Grant/Research Support)

